# Immunosuppressant Peptide Abu-TGIRIS-Abu-NH_2_ and its Application for Treatment of Multiple Sclerosis

**DOI:** 10.1007/s12668-018-0513-8

**Published:** 2018-03-02

**Authors:** Valery I. Turobov, Viatcheslav N. Azev, Alexei B. Shevelev, Natalia V. Pozdniakova, Yulia K. Biryukova, Arkady N. Murashev, Valery M. Lipkin, Igor P. Udovichenko

**Affiliations:** 1 Laboratory of Protein Chemistry, Branch of Shemyakin and Ovchinnikov Institute of Bioorganic Chemistry, Russian Academy of Sciences, Prospekt Nauki, 6, Pushchino, 142290 Russia; 2 Emanuel Institute of Biochemical Physics, Moscow, Russia; 3 Blokhin Russian Cancer Research Centre, Moscow, Russia; 4 Pushchino Research Center, Russian Academy of Sciences, Pushchino, Russia; 5 Pushchino State Institute of Natural Sciences, Pushchino, Russia

**Keywords:** Immunocortin, Multiple sclerosis, Peptide, Drugs, Immunosuppressants, ACTH

## Abstract

Immunosuppressant peptide immunocortin for the first time was described in 1993. It corresponds to residues 11–20 of human Ig heavy chain (conserved motif of V_H_ domain). There are no data about production of immunocortin by proteolysis of Ig in vivo. Synthetic immunocortin in concentration ~ 10^−9^ M suppresses phagocytosis in peritoneal macrophages, ConA-dependent blast transformation of rat lymphocytes, exhibits ACTH-like neurotropic activity and was suggested as a potential drug for treatment of a multiple sclerosis (MS). Here, we report a sequence and method of synthesis of Abu-TGIRIS-Abu-NH_2_ (Abu, alpha-aminobutyric acid), an artificial analogue of immunocortin. Biological trials of peritoneally injected Abu-TGIRIS-Abu-NH_2_ gave an evidence of its better efficacy versus immunocortin in a test for suppression of the experimental autoimmune encephalomyelitis (EAE) in Dark Agouti (DA) rats.

## Introduction

Multiple sclerosis (MS) is a severe neurological condition of autoimmune origin with a worldwide distribution. The 2012 MS prevalence in the USA was 149.2 per 100,000 individuals (95% confidence interval 147.6–150.9). Prevalence was consistent over 2008–2012 [1]. Current costs for most MS disease-modifying therapies in the USA exceed $70,000 a year [2]. Detailed mechanism of MS onset remains unknown. The key reason of motor functions in a patient is impairment of myelin sheath in the central nervous system. Myelin is composed with myelin basic protein (MBP), proteolipid protein (PLP), myelin-associated glycoprotein (MAG), and myelin oligodendrocyte glycoprotein (MOG) [3]. On the basis of clinical manifestation, MS is divided into four categories: relapsing-remitting MS (RRMS)—85% from registered MS cases; primary progressing MS (PPMS)—10% cases; SPMS—secondary progressing MS (develops in patients with RRMS); and progressing-remitting MS (PRMS)—5% cases [4]. Both T- and B-lymphocytes are involved to MS initiation and progression. B-lymphocytes infiltrated to cerebrospinal liquid through hematoencephalic barrier are responsible for oligoclonal antibodies some of which exhibit low affinity to myelin [5]. These B-lymphocytes are prone to an enhanced synthesis of a lymphoproliferative cytokine GM-CSF [6].

There are several types of medicines applied for abatement of MS symptoms:

1. Massive intravenous injections of donor Ig (IVIG);
2. Non-specific cytostatic agents the same ones that are used for treatment of tumors and rheumatoid arthritis;
3. Steroid immunodepressants and peptide immunodepressants derivatives of adrenocorticotropin (ACTH);
4. Interferons β-1a, β-1b, and γ (including their modified forms with prolonged action);
5. Modulators of S1P-receptors, e.g., Fingolimod (2-amino-2-[2-(4-octylphenyl)ethyl]propan-1,3-diol and others;
6. Т cell vaccines intended to activation or suppression of antigen-dependent recognition of myelin proteins;
7. Antagonists of B cell receptors e.g., humanized monoclonal antibodies to CD20, receptors of IL-2 and Fcγ;
8. Humanized monoclonal antibodies to GM-CSF;
9. Peptides mimicking MBP and/or other myelin proteins;
10. Combinations of abovementioned substances.

The most popular medicines for treatment of MS are IFNβ-1a, IFNβ-1b, glatiramer acetate, fingolimod, natalizumab, and dimethyl fumarate [7]. Anti-MS immonobiological preparations passing the stage of clinical trials are Ocrelizumab (antibody to B cell antigen CD20) [8]; Natalizumab (Tysabri®) (an antibody to integrin α4) [9]; Daclizumab (an antibody against α-subunit of IL-2R [10]). Mitoxantrone (Novantrone®) is a cytostatic agent with a certain tropism to immune system cells [11]. Fingolimod by Gilenya is a non-selective agonist of sphingosine-1-phosphate receptor FTY720 (S1P) [12]. Fingolimod stops infiltration of lymphocytes through blood-brain barrier to the central nervous system. Specific action of Glatiramer acetate (Copaxone) by Teva Pharmaceuticals, a stochastic polymer of four amino acids overrepresented in myelin is assigned to specific blocking anti-myelin antibodies [3]. Glatiramer acetate has the greatest demand at the market, since it does not exhibit unfavorable side effects although its clinical efficacy is disputable. All abovementioned immune-modifying drugs are inefficient against primary and secondary non-remitting (progressive) forms of MS which are less prevalent than RRMS but often exhibit high severity [13].

Abovementioned data illustrate a high actuality of designing novel immunosuppressant for treatment of MS particularly its progressing forms. ACTH efficiently suppresses MS in animal models and prevents anti-myelin lymphocytes in vitro. However, clinical application of ACTH as immunosuppressant is precluded with multiple, severe, and poor predictable side effects (e.g., vascular dystonia, systemic hormonal disorders, kidney dysfunction, mental dysfunction). Some endogenous peptides from human and rat peptides exhibit immunosuppressant activity similar to ACTH but are free from its side effects [14]. These are immunocortin (VKKPGSSVKV) (13), tuftsin (res. 289-292 of γ-chain), rigin (341-344), immunorphin (364-373), and peptide p24 (335-358)). Immunocortin is derived from Ig VH-fragment (11–20 of human γ- or μ-chain); it exhibits immunosuppressing properties. However, practical application of immunocortin is precluded by its low stability in vivo.

Here, we report biological trials of the synthetic artificial peptide Abu-TGIRIS-Abu-NH_2_ obtained by a computational modification of immunocortin. This peptide shorter and contains terminal residues of α-aminobutyrate. This theoretically makes Abu-TGIRIS-Abu-NH_2_ more stable in vivo and facilitates chemical synthesis. Objective of this study was testing of anti-MS efficacy of Abu-TGIRIS-Abu on experimental autoimmune encephalomyelitis (EAE) model in rats in comparison with a shortened analogue of immunocortin Abu-SSVKVs-Abu-NH_2_.

## Methods

### Peptide Synthesis, Purification, and Analysis

Peptides Abu-TGIRIS-Abu-NH_2_ and Abu-SSVKVS-Abu-NH_2_ were prepared by solid-phase peptide synthesis (SPPS) technique, using Fmoc/tBu-protected amino acids as described elsewhere [15]. Purification of the crude peptides was carried out using RP HPLC (C_18_ 250 × 50 mm Vydac column, isocratic elution with 10% acetonitrile, and 0.1% TFA in water, UV detection at 226 nm). Homogeneity of the peptides was confirmed using RP HPLC (C_18_ 250 × 4.6 mm Vydac column, gradient elution 5 to 30% acetonitrile/0.1% aqueous TFA over 25 min, UV detection at 226 nm). The identity of purified peptides was confirmed using mass spectrometry (microTOF-Q, Bruker Daltonics GmbH).

### Animals

Dark Agouti (DA) rats (DA/ZFV Crl BR breeding stock was purchased from Charles River Co., Sulzfeld, Germany) were bred at the Branch of Shemyakin, and Ovchinnikov Institute of Bioorganic Chemistry, Pushchino, Russia. Animals were kept at the animal facility under the climate-controlled conditions with 12 h light/dark cycles and fed with food, and water provided ad libitum.

The study was carried out in accordance with the Institutional Animal Care rules, and User Program, Federal Guidelines SP 2.2.1.3218-14 (Russian Federal Service for Surveillance on Consumer Rights Protection, and Human Wellbeing, 2014); the Guide for the Care, and Use of Laboratory Animals: Eight Edition (National Research Council, 2011), Guidelines for the Care, and Use of Mammals in Neuroscience, and Behavioral Research (National Research Council, 2003), the Directive 2010/63/EU of the European Parliament, and of the Council of 22 September 2010 on the protection of animals used for scientific purposes. The protocol no. 521/16 was approved by the Institutional Committee for Ethics on Animal Care, and Use at the Branch of Shemyakin, and Ovchinnikov Institute of Bioorganic Chemistry, Russian Academy of Sciences (Pushchino, 142290, Russia).

### Biological Trials

Spinal cord homogenate was prepared from non-linear rats as described formerly [16]. Thirty DA rats, weighing 220–250 g, were injected with syngeneic spinal cord homogenate in incomplete Freund’s adjuvant (1:1 *w*/*v*) into hind footpads (100 μl/footpad). The day after immunization, the rats were divided into three groups, 10 animals each. Abu-TGIRIS-Abu and Abu-SSVKVS-Abu peptides were injected intraperitoneally (i.p.) to each rat from one of two experimental groups, respectively. Placebo (sterile non-pyrogenic saline) was administrated to 10 animals of the control group. Further, the rats were treated by the same scheme daily for 18 days. The daily dosage of the peptide was 400 μg/kg in total volume 100 μl per animal in a normal saline. All rats were weighed daily and examined for clinical signs of EAE. The clinical grading was used as follows: 0, asymptomatic; 1, loss of tail tonicity; 2, impaired righting reflex; 3, partial paralysis; 4, complete paralysis; 5, moribund or dead animals. Clinical signs of a lower severity than typically observed were scored 0.5 lower than the grade indicated. Typical EAE onset was observed 8 to 10 days after immunization with peak of disease from 11 to 14 days after immunization. The peak of the disease lasted from 2 to 3 days.

### Statistical Analysis

Data from EAE experiments were evaluated as the mean EAE score ± SEM and with Friedman’s ANOVA. Statistical analysis was performed using IBM SPSS Statistics v.22.

## Results

The peptide Abu-SSVKVS-Abu-NH_2_, as a prototype, was formerly derived from immunocortin by reducing its length and introducing Abu residues at both ends. The peptide Abu-TGIRIS-Abu-NH_2_ was constructed from Abu-SSVKVS-Abu-NH_2_ prototype by the rational design methodology in accordance with the similarity between amino acid residues based on the analysis of the surroundings of each residue in primary structures of native sequences [17] (substitutions: S2 → T, S3 → G, V4 → I, K5 → R, V6 → I).

### Peptide Synthesis

When applying Boc/Bzl SPPS methodology for the preparation of Abu-TGIRIS-Abu-NH_2_ peptide, we encountered a quite an unusual problem of a slow acylation of Arg residue with various activated derivatives of isoleucine. Therefore, we attempted to employ a second available Fmoc/tBu SPPS methodology in connection with possibilities of using an alternative polymer together with a wider range of activated protected isoleucine derivatives available in Fmoc SPPS. Standard tert-butyl ether-type protective groups were employed for protection of side chains of Ser and Thr. The side chain of Arg was protected with 2,4,6,7-pentamethyl-dihydrobenzofuran-5-sulfonyl moiety (Pbf).

**Fig. 1:**
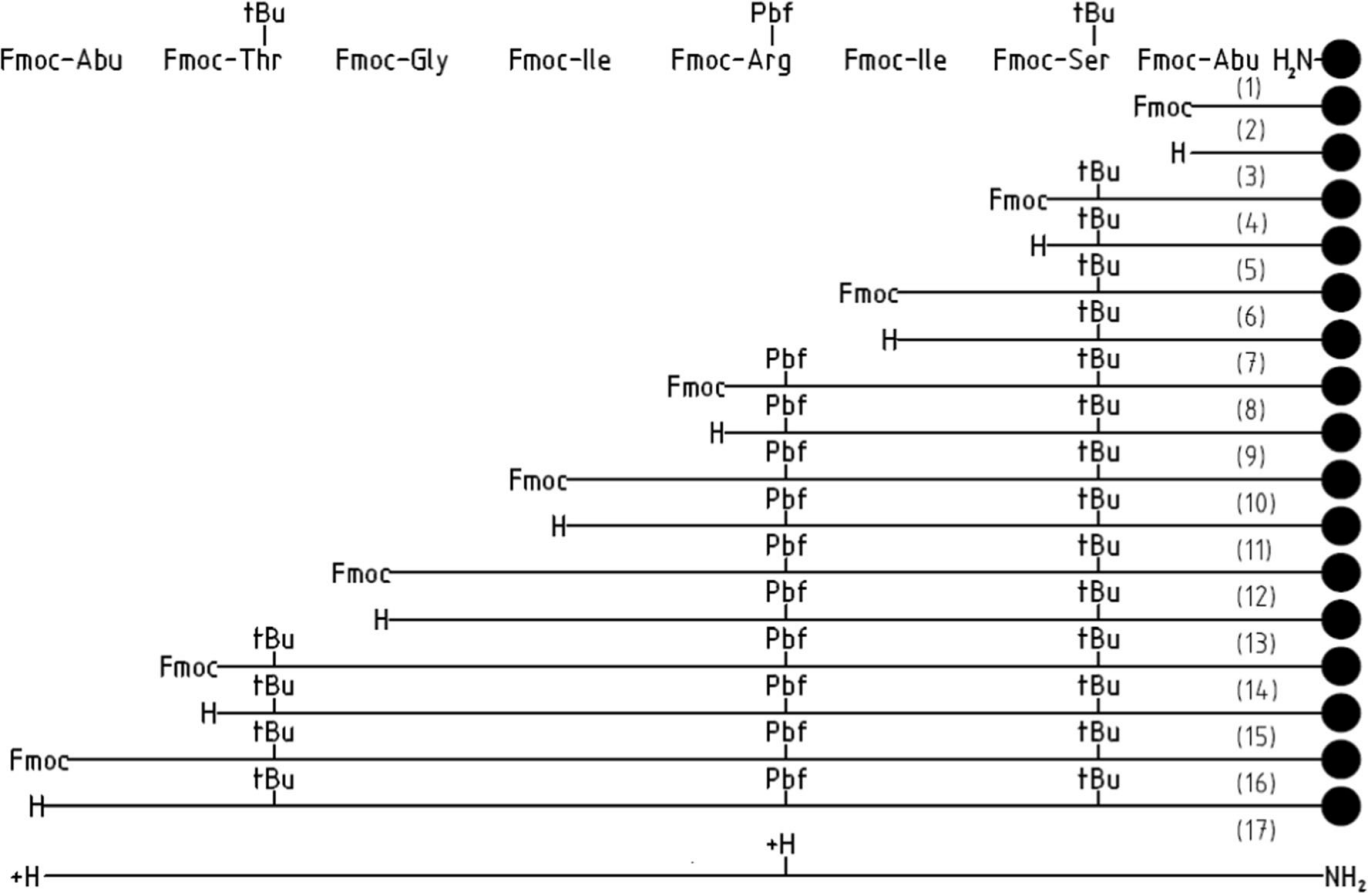
Solid phase synthesis of Abu-TGIRIS-Abu-NH_2_ by using Fmoc/tBu-protected amino acid derivatives. Polystyrene resin modified with Rink-amide linker is designated as a solid disk. Growing chain is denoted as a straight line. Actual transformations for conversion into functional moieties are shown nearby N-terminus of the growing chain (on the left). Numbers in the column on the right correspond to the stages of the synthesis and to chemicals for transformation of the peptidyl-polymer. (1), (3), (5), (7), (11), (13), (15): Fmoc-Xaa-OH (2.9 eq.), TBTU (3 eq.), HOBt (3 eq.), NMM (3 eq.), DMF; (2), (4), (6), (8), (10), (12), (14), (16): 4-MePip/DMF 20/80; (9) Fmoc-Ile-F; (17) TFA, H_2_O 95/5 *v*/*v*

Synthesis of Abu-TGIRIS-Abu (Fig. 1) begins from a condensation of Fmoc-protected α-aminobutyrate with amino group of the polystyrene resin modified with Rink-linker (4-[(2,4-dimethoxyphenyl)(Fmoc-amino)methyl]phenoxyacetic acid). Initially O-(Benzotriazol-1-yl)-N,N,N′,N′-tetramethyluronium tetrafluoroborate (TBTU) was used as a combining agent. After each condensation, extent of conversion of the amino group acylation was controlled qualitatively with ninhydrin test [18] and quantitatively with picric acid test [19]. Analysis demonstrated that condensation of hydroxybenzotriazolyl-activated ester of Fmoc-Ile-OBt (obtained in the reaction with TBTU) with the peptidyl-polymer Arg(Pbf)-Ile-Ser(^t^Bu)-Abu-Rink-PS is as slow as in the case of Boc/Bzl-protected amino acid derivatives. After experimental screening of various Fmoc-Ile activated derivatives (Table 1), we have discovered that fluoro-anhydride derivative (Fmoc-Ile-F) provided almost complete conversion of the immobilized peptidyl-polymer.

**Table 1:** Optimization of protocol for synthesis of Abu-TGIRIS-Abu-NH_2_ by using different Fmoc/tBu-protected amino acid derivatives^a^

Conditions			Result	
Xaa	Chemical	Solvent	Ninhydrin test %^b^	Picric test %^c^
Abu	TBTU	DMF	> 98	99.6
Ser(^t^Bu)	TBTU	DMF	> 98	99.2
Ile	TBTU	DMF	> 98	99.3
Arg(Pbf)	TBTU	DMF	> 98	99.7
Ile	TBTU	DMF	< 85	12.3
Ile	TBTU	DMA	< 85	16.8
Ile	TBTU	NMP	< 85	21.2
Ile	TBTU	4 M KSCN in DMF	< 85	24.8
Ile	TBTU	NMP	< 85	25.5^d^
Ile	(Fmoc - Ile)2O	NMP	< 85	34.4
Ile	HATU	NMP	< 85	47.9
Ile	Fmoc-Ile-F	NMP	> 85	89.1
Ile	Fmoc-Ile-F	NMP	> 98	98.6^d^
Gly	TBTU	DMF	> 98	99.4
Thr(^t^Bu)	TBTU	DMF	> 98	99.5
Abu	TBTU	DMF	> 98	99.5

^а^Yield of acylation after repetitive condensation

^b^Quantitative estimate

^c^Arithmetic mean value of two reactions

^d^After three subsequent condensations

It should be noted that an attempt to vary the solvent composition has not lead to any improvement in the condensation rates. Moreover, involvement of 4 M KSCN in DMF as a “chaotropic additive” does not affect rate of Arg condensation with Fmoc-Ile-OBt. This additive is commonly used for preventing aggregation of the polymer matrix (phase collapse). This provides evidence that slow acylation of Arg is likely caused by steric hindrance not by the phase collapse. This hypothesis is in a good agreement with the fact that Fmoc-Ile-F having the smallest exiting group provides the highest rate of Arg conversion. A symmetric anhydride (Fmoc-Ile)_2_O exhibits a moderate reactivity. Noteworthy, Fmoc moiety is bulkier than Boc and hence contributes more to steric limitation of interaction of the reactive centers.

Further condensations of the protected amino acid derivatives were carried out using TBTU as a coupling reagent. Removal of the protection groups and extraction of the peptide from the polymer was carried out with TFA together with water as a cation scavenger. Reverse phase HPLC demonstrated the presence of the product of interest in the reaction mixture (~ 35%). Mass spectrometry allowed identification of the product of interest and a number of impurities with MW 1067.58 a.u.m (+Pbf), 773.49 (Arg → Orn), and 1016.61 (Arg → Orn(Abu-Thr-Gly)). Transformation of Arg to ornithine gives an evidence of acylation of guanidine moiety of Arg residue with highly reactive acylating agents. Triplicate chromatographic separation of the reaction mixture provides obtaining of the product of interest with a theoretical yield ~ 12%.

The described method of solid-phase peptide synthesis allowed to produce Abu-TGIRIS-Abu-NH_2_ in amount required for biological trials (~ 100–500 mg). Likely to Boc/Bzl-methodology, acylation of Arg(PG)-Ile-Ser(PG)-Abu-polymer limits the overall yield and rate of the reaction. Apparently, this product immobilized on polystyrene accepts a specific conformation masking the amino group from bulky electrophilic agents.

As it was mentioned, Abu-TGIRIS-Abu-NH_2_ peptide was also synthesized using Boc/Bzl methodology. In this case, the chemical yield of the product was lower probably due to the fact of a slow rate of Arg acylation. It should be also noted that an attempt of preparation of Boc-Ile-F derivative in order to solve the slow rate acylation problem has failed in our hands.

The prototype peptide, Abu-SSVKVS-Abu-NH_2_, was synthesized using both solid-phase methodologies in higher yields. Each scheme was reproduced in triplicate. Efficiency of Boc/Bzl and Fmoc/tBu-methodology for synthesis of Abu-TGIRIS-Abu-NH_2_ and Abu-SSVKVS-Abu-NH_2_ peptides is shown in Table 2. The comparison unambiguously proves preference of Fmoc/tBu-methodology for producing immunosuppressant Abu-TGIRIS-Abu-NH_2_ and Abu-SSVKVS-Abu-NH_2_ peptides for biological trials.

**Table 2:** Comparison of yields and purity of immunosuppressant Abu-TGIRIS-Abu-NH_2_ and Abu-SSVKVS-Abu-NH_2_ peptides

SPPS methodology	Peptide			
	Abu-TGIRIS-Abu-NH_2_		Abu-SSVKVS-Abu-NH_2_	
	Average yield, %	Final purity (HPLC), %	Average yield, %	Final purity (HPLC), %
Boc/Bzl	7.1 ± 0.8*	96.7	19.1 ± 1.1*	97.2
Fmoc/tBu	12.2 ± 1.1*	97.1	22.3 ± 0.8*	97.4

*Each value shown is the mean ± SEM (*n* = 3)

The purification of the immunosuppressant peptides was carried out as described in “Methods” section. Experimentally determined molecular mass of Abu-TGIRIS-Abu-NH_2_ was 815.4 a.u.m. and Abu-SSVKVS-Abu - 775.3 a.u.m.

### Efficacy of Immunocortin Therapy In Vivo

**Fig. 2:**
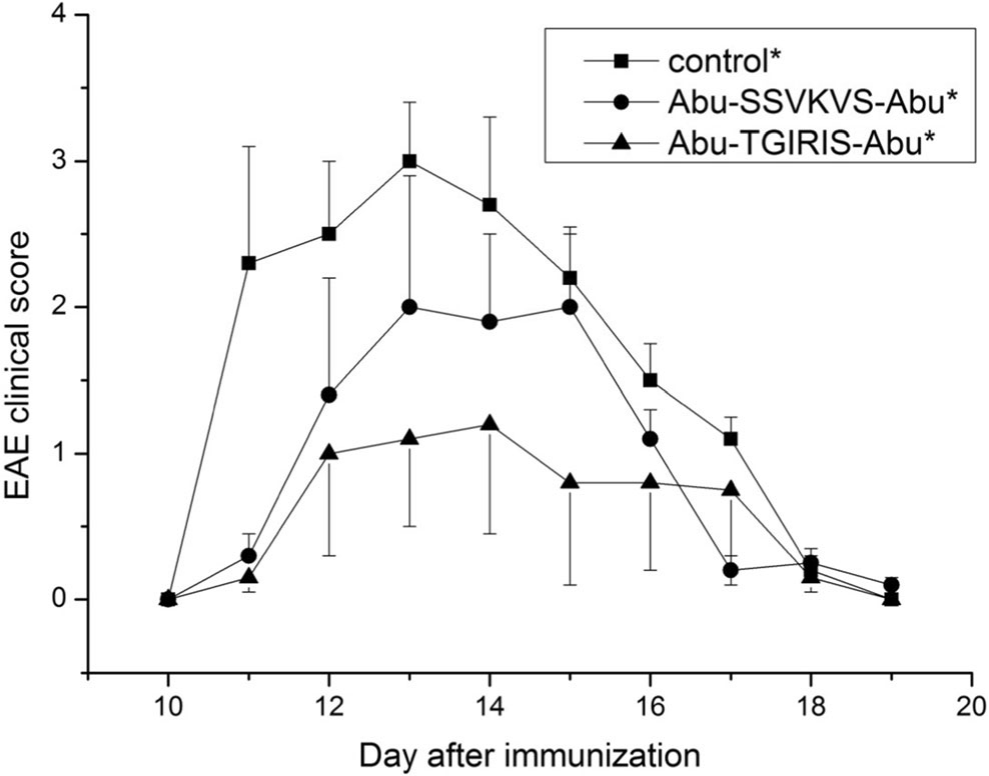
Biological trials of immunosuppressant peptides Abu-TGIRIS-Abu-NH_2_ and Abu-SSVKVS-Abu-NH_2_ in vivo on EAE model in DA rats. Abscises axis, time after immunization, days; ordinate axis, average severity of EAE in points. Control group (*n* = 10) was injected with placebo (normal saline). Experimental groups (*n* = 10 each) were administrated with immunosuppressants Abu-SSVKVS-Abu-NH_2_ and Abu-TGIRIS-Abu-NH_2_ in dosage 400 μg/kg. Each value shown is the mean ± SEM (*n* = 10). *p < 0.05, by Friedman’s ANOVA

Immunosuppressant peptides Abu-TGIRIS-Abu and Abu-SSVKVS-Abu were tested for suppressing EAE symptoms in DA rats as described in “Methods” section. Evident signs of paralysis were found in the EAE rats from 11 to 19 days after immunization (Fig. 2). The acute phase of the condition lasted for 2–3 days in period 11–14 days after immunization. Both peptides showed the ability to reduce symptoms of EAE, and Abu-TGIRIS-Abu-NH_2_ was more effective.

## Conclusion

Comparison of Fmoc/tBu- and more traditional Boc/Bzl-methodology of the solid-phase peptide synthesis demonstrated unambiguous preference of the first one for synthesis of the proposed immunosuppressant peptides Abu-TGIRIS-Abu-NH_2_ (1.7 times) and Abu-SSVKVS-Abu-NH_2_ (1.2 times). Involvement of this method provided sufficient amount of pure peptides (100–500 mg) with satisfactory purity (> 97%).

Abu-TGIRIS-Abu-NH_2_ demonstrated higher efficacy than Abu-SSVKVS-Abu-NH2 in the test for suppressing EAE symptoms in DA rats. Intraperitoneal injection of Abu-SSVKVS-Abu-NH_2_ caused delay of the acute phase of the paralysis, whereas Abu-TGIRIS-Abu-NH_2_ administrated with the same scheme completely abolished the most severe symptoms.

Abu-TGIRIS-Abu-NH_2_ should be considered as a promising agent for treatment of multiple sclerosis and probably other autoimmune conditions, e.g., rheumatoid arthritis. Likely to all other drugs practically used for treatment of MS (except glatiramer acetate mechanism of which is disputable), Abu-TGIRIS-Abu-NH_2_ does not affect antigen-dependent mechanisms of the disease. However, it may have relatively low side effect due to expected low penetration ability and rapid degradation. Extensive toxicological trials are required for assessing prospects of Abu-TGIRIS-Abu-NH_2_ as a candidate drug.
